# Do probiotics promote oral health during orthodontic treatment with fixed appliances? A systematic review

**DOI:** 10.1186/s12903-020-01109-3

**Published:** 2020-04-25

**Authors:** Riham Hadj-Hamou, Abiola C. Senok, Athanasios E. Athanasiou, Eleftherios G. Kaklamanos

**Affiliations:** 1 Specialist Orthodontist, Dubai, United Arab Emirates; formerly Hamdan Bin Mohammed College of Dental Medicine, Mohammed Bin Rashid University of Medicine and Health Sciences, Dubai, United Arab Emirates; 2 College of Medicine, Mohammed Bin Rashid University of Medicine and Health Sciences, Dubai, United Arab Emirates; 3 Department of Dentistry, European University Cyprus, Nicosia, Cyprus; 4 Hamdan Bin Mohammed College of Dental Medicine (HBMCDM), Mohammed Bin Rashid University of Medicine and Health Sciences, Building 34, Dubai Healthcare City, Dubai, United Arab Emirates

**Keywords:** Probiotics, Gingivitis, Caries, White spot lesions, Oral health, Orthodontic treatment

## Abstract

**Background:**

Treatment with fixed orthodontic appliances has been associated with significant biofilm accumulation, thus putting patients at a higher risk of oral health deterioration. The use of probiotics has been proposed to be useful in the prevention or treatment of oral pathologies such as caries and diseases of periodontal tissues. Our aim was to investigate the effects of probiotic use on inflammation of the gingival tissues and the decalcification of the enamel in patients being treated with fixed orthodontic appliances.

**Methods:**

We searched without restrictions 8 databases and performed hand searching until September 2019. We searched for randomized controlled trials (RCTs) evaluating whether individuals with fixed orthodontic appliances benefit from probiotic treatment in terms of the inflammation of the gingivae and decalcification of the enamel. Following the selection of studies and the extraction of pertinent data, we appraised the risk of bias and the confidence in the observed effects based on established methodologies.

**Results:**

From the final qualifying studies, three did not show any statistically significant effect on gingival inflammation after probiotic administration of up to 1 month. Similarly, non-significant differences were noted in another study regarding white spot lesions development (mean administration for 17 months). No adverse effects were reported and the level of evidence was considered moderate.

**Conclusions:**

Supplementation of orthodontic patients with probiotics did not affect the development of inflammation in the gingivae and decalcification in the enamel. Additional RCTs, with longer intervention and follow-up periods, and involving different combinations of probiotic strains are required.

**Trial registration:**

PROSPERO (CRD42018118008)

## Background

Orthodontic procedures aim to establish a healthy, functional and appealing occlusion that is in balance with facial aesthetics [1]. However, they have been associated with particular oral hygiene challenges when fixed orthodontic appliances are involved, as these act as plaque traps and render satisfactory mechanical oral hygiene laborious [2]. The continued biofilm accumulation could lead to inflammatory changes with concomitant anaerobic shift in the oral microbiota, which can be clinically recognized as gingival bleeding [3, 4]. Moreover, whenever a cariogenic environment is favoured, enamel decalcification can also be observed [5, 6]. As these alterations in the oral environment might be detectable even after 2 years after the removal of orthodontic appliances [7, 8], meticulous oral hygiene, as well as oral health maintenance are considered paramount for the favourable outcome of orthodontic treatment [2].

Probiotics are defined as “live microorganisms which when administered in adequate amounts confer a health benefit on the host” [9]. Since the pathogenesis of caries and the diseases of the periodontal tissues has been associated with alterations in composition of oral microbiome and biofilm formation, the administration of probiotic strains has been proposed to be useful in their prevention and treatment [10–12]. In vitro studies using specific probiotic strains have demonstrated beneficial effects against oral pathogens [13–20]. However, the clinical effectiveness of administering probiotics to positively affect oral health remains undetermined. Whilst findings from an increasing number of studies supports the use of probiotic strains in the prevention or treatment of gingivitis and periodontitis, other trials have failed to show similar effects [21, 22]. Moreover, though the consumption of probiotics has been proposed as a supporting measure for caries prevention based on surrogate markers [23, 24], insufficient information on actual clinical benefits exists [21].

### Objective

As treatment with fixed orthodontic appliances has been associated with significant biofilm accumulation, thus putting patients at a greater risk of developing caries and gingivitis [3–6], probiotics could be of benefit. However, studies solely targeting on the clinical effects on orthodontic patients are limited and have not, so far, been reviewed in an evidence-based manner. Our aim was to systematically assess the available evidence from randomized Clinical Trials (RCTs) on whether probiotics reduce gingival inflammation and enamel demineralization development in patients under treatment with fixed orthodontic appliances.

## Methods

### Protocol development and registration

For the development of the review protocol we adhered to relevant guidelines [25, 26]. The protocol was subsequently listed in the PROSPERO database (CRD42018118008). Being a systematic review, ethical approval was not required for this study.

### Eligibility criteria

The criteria were formed on the PICOS basis. For the participants domain we aimed at including studies involving healthy orthodontic patients without age restrictions. Studies on subjects with syndromic or other anomalies of the craniofacial region, individuals with systematic diseases, as well as patients using antibiotics or antimicrobial agents were not considered. Study participants should receive probiotics of any type and be compared to individuals receiving placebo or no intervention at all. The outcomes considered included clinical measurements on gingival inflammation and enamel demineralization development. Plaque measurements were not considered, as reductions in plaque do not always directly reflect benefits in oral health, which is the primary goal of the intervention [27]. Person reported outcomes (preferences, experiences, quality of life, satisfaction, etc.), as well as adverse effects and economic evaluation data were also of interest. Only RCTs were eligible for inclusion. Human studies that did not evaluate clinical outcomes, animal studies, studies without control groups and reviews were excluded (Supplementary Table 1).

### Information sources and search strategy

We searched without restrictions the whole content in eight databases, from the beginning of the period covered in each database and up until September 2019 (MEDLINE, CENTRAL, Cochrane Systematic Reviews, Scopus, Web of Science, Arab World Research Source, Clinical Trials registry and ProQuest Dissertations and Theses Global database). We used detailed strategies, developed and customized by one of the researchers (RHH) without placing restrictions on language (Supplementary Table 2). The list of references in the included studies were scanned and we planned to contact their corresponding authors in case we needed additional information.

### Study selection of studies and data extraction

The list of records produced by the search was assessed by two researchers (RHH and EGK) independently, in a non-blinded manner, but kappa scores for the extent of agreement were not calculated, as it is not recommended [26]. The same investigators performed data extraction, using pre-designed data collection forms to record the following: duration of follow-up; individual characteristics (inclusion/exclusion criteria, number, age, gender, possible dropouts); interventions (experimental and placebo/control groups; specific details on the probiotic product used, dosage and duration of administration); details on outcomes assessed; and if available, data on patient reported outcomes, adverse effects and economic evaluation data. Finally, additional information was extracted, where possible, concerning a priori sample power analysis and the assessment of reliability. Disagreements were settled by deliberation with a third researcher (AEA).

### Risk of bias in individual studies

Judgements on the risk of bias, on domain and study levels, were completed independently by EGK and ACS, using established methodology [26]. Disagreements were settled as stated previously.

### Summary measures and synthesis of results

Although synthesis of the results (using either the Weighted Mean Differences or the Standardized Mean Differences) was planned according to the research protocol, it was not, in the end, carried out due to the lack of adequate data as well as differences in the retrieved studies.

### Risk of bias across studies and additional analyses

For the reasons stated above, although planned, we were not able to conduct any exploratory subgroup analyses, in addition to analyses for “small-study effects” and publication bias [26]. Despite the lack of extensive information, the quality of evidence was assessed following Guyatt et al. [28] in order to adopt a structured and transparent approach in formulating an interpretation of the evidence.

## Results

### Study selection

**Fig. 1:**
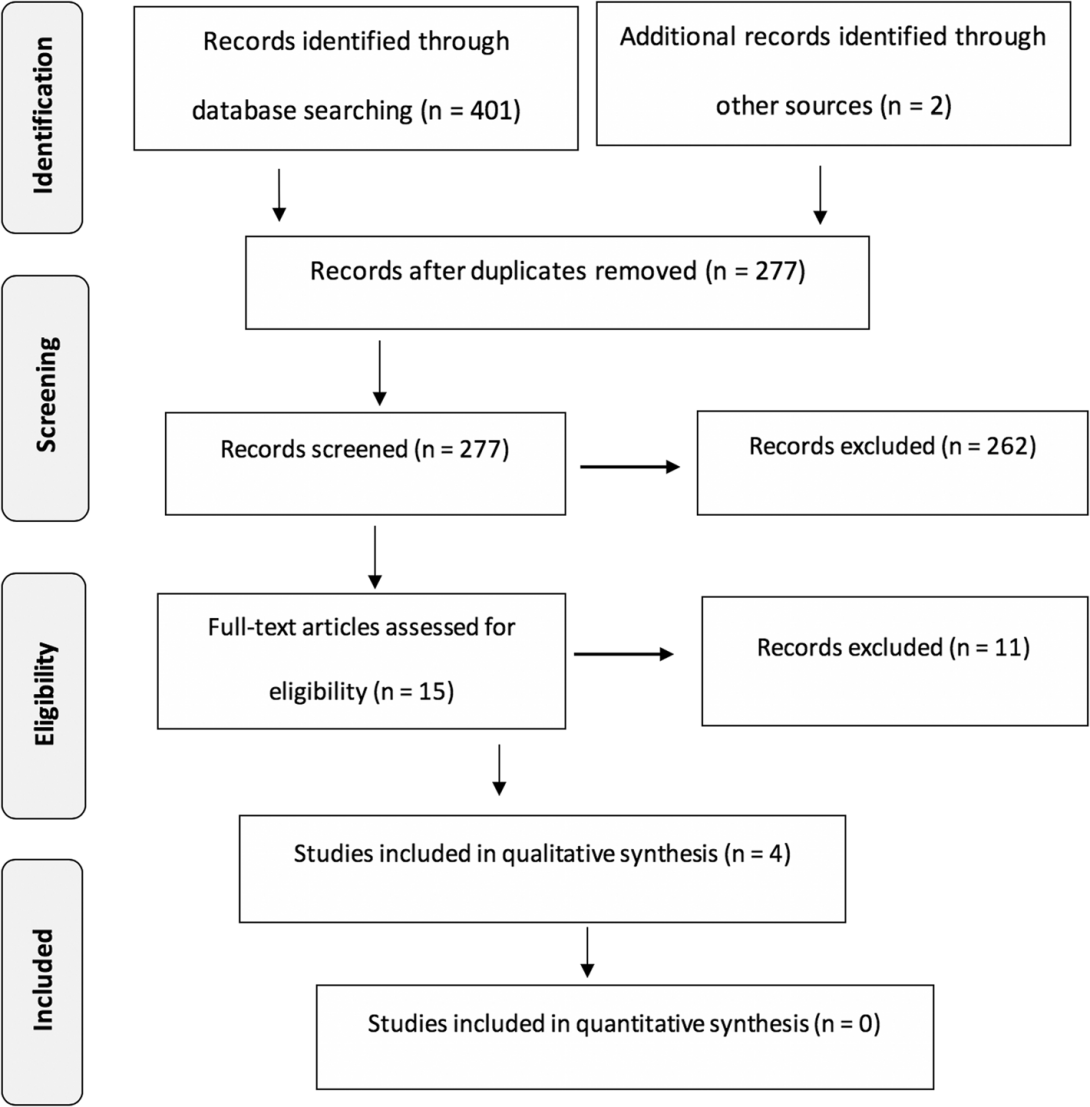
Flow diagram of the records through the reviewing process

From the 403 records initially identified, we excluded 126 as duplicates, 262 based on title and/or abstract, and 11 more after reading the full paper (Fig. 1) (Supplementary Table 3). Finally, four RCTs were included [29–32] (Tables 1 and 2).

**Table 1:** General characteristics of the studies included in the systematic review

Study	Intervention characteristics	Outcomes assessed	Others
**Benic [2019]** [29] University of Otago New Zealand	**EG:** Lozenges [*Streptococcus salivarius* M18 - 3 × 10^9^ CFU/lozenge] **PG:** Lozenges without active bacteria **Administration:** 1 month **Dosage:** 2 lozenges/d	**Gingival inflammation:** Gingival index [33] **Adverse effects**	**Sample size calculation:** Yes, but not for GI **Reliability of measurements:** Not reported
**Gizani et al. [2015]** [30] University of Athens Greece	**EG:** Lozenges [*Lactobacillus reuteri DSM 17938* and *Lactobacillus reuteri ATCC PTA* 5289–10^8^ bacteria of each strain] **PG:** Identical lozenges without active bacteria **Administration:** from the time of enrollment until debonding (mean ± SD: 17.0 ± 6.8 months) **Dosage:** 1 lozenge/d; after brushing, before bedtime	**Enamel demineralization:** Gorelick et al. [34] White Spot Lesion Index assessed photographically **Adverse effects**	**Sample size calculation:** Yes **Reliability of measurements:** Yes
**Habib [2016]** [31] University of Toronto Canada	**EG:** Lozenges [*Streptococcus salivarius* K12*, Lactobacillus paracasei, Lactobacillus plantarum, Lactobacillus acidophilus, Lactobacillus salivarius, Lactobacillus reuteri* - 3 × 10^9^ CFU/lozenge] **PG:** Identical lozenges without active bacteria **Administration:** 4 weeks **Dosage:** loading dose - 2 lozenges, 2 times/d (after breakfast and after dinner) for 1w; maintenance dose; 2 lozenges, 1/d (after breakfast) for next 3 w	**Gingival inflammation:** Modified Gingival Index [35] **Adverse effects**	**Sample size calculation:** Yes **Reliability of measurements:** Yes
**Kohar et al. [2015]** [32] Trisakti University Indonesia	**EG**_**1**_**:** Lozenges [*Lactobacillus reuteri* - 2 × 10^8^ CFU/lozenge] **EG**_**2**_**:** Drink [*Lactobacillus casei* strain Shirota - 6.5 × 10^6^/bottle] **CG** **Administration:** 2 weeks **Dosage:** 1 lozenge or 65 ml/d, at least 1 h after lunch	**Gingival inflammation:** Papillary Bleeding Index [36]	**Sample size calculation:** Not reported **Reliability of measurements:** Not reported

*CG Control group, without placebo administration, EG Experimental group, h hour, PG Placebo group, w week*

**Table 2:** Participant characteristics in included studies

Study	Inclusion and exclusion criteria	Analyzed sample [age; gender]
**Benic [2019]** [29] University of Otago New Zealand	**Inclusion Criteria:** Presence of at least 20 natural teeth; stainless steel brackets **Exclusion Criteria:** Systemic disease; lingual braces; living in a non-fluoridated area; periodontal disease; taking antibiotics; using non-fluoride/antibacterial toothpaste; dental fluorosis; smoking; using powered toothbrushes; lactose intolerance; allergy to dairy products; physical inability to brush	**Age [range]:** 10-30y **EG:** 32 [20F, 12 M] **PG:** 32 [21F, 11 M] **Missing data:** No
**Gizani et al. [2016]** [30] University of Athens Greece	**Inclusion Criteria:** Fixed appliances on at least eight maxillary front teeth (incisors, cuspids, and premolars); expected duration of Tx 7–24 m **Exclusion Criteria**: Individuals under treatment with systemic or local antibiotics up to two weeks before starting the study	**Age [mean ± SD]:** 15.9 ± 3.9y **EG:** 42 (23F, 19 M); **PG**: 43 (33F, 10 M) **Missing data:** 9 [errors in follow-up photos]
**Habib [2016]** [31] University of Toronto Canada	**Inclusion Criteria:** Healthy; aged 11–18 y; mild to moderate gingivitis; fixed appliances on at least 20 teeth for at least 5 m; complete eruption of teeth #16, 21, 23, 36, 41, 43; no active caries; no use of antimicrobial mouth rinses, probiotics, antibiotics or anti-inflammatory drugs within 1 m before the trial; undergone standard orthodontic bonding procedure **Exclusion Criteria:** Inability to consent or communicate fluently in English; allergies or sensitivity to lozenge ingredients; immunocompromised; major underlying medical condition or ENT problems; pregnancy; smoking, alcohol consumption; oral diseases or conditions; surgery within the past 45d or the next 90d; use of antibiotics, anti-inflammatory drugs, ongoing or use of probiotics within the past 1 m; nausea, fever, vomiting, bloody diarrhea or severe abdominal pain within the past 1 m; molar bands	**Age [mean ± SD; range]:** 15.69 ± 1.70y; 11-18y **EG:** 29 [15.75 ± 1.67y; 13F, 16 M] **PG:** 29 [15.64 ± 1.75y; 20F, 9 M] **Missing data:** 1/group [lost from to follow-up]
**Kohar et al. (2015)** [32] Trisakti University Indonesia	**Inclusion Criteria:** Healthy; no medication; aged 18-25y; fixed appliances Tx for at least 1y **Exclusion Criteria**: Using xylitol gums, mouthwashes, systemic antibiotics; smokers; pregnancy; topical fluoride treatment	**Age [range]:** 18-25y **EG**_**1**_**:** 10; **EG**_**2**_**:** 10; **CG:** 10 **Missing data:** No

*CG* Control group, without placebo administration, *d* days, *EG* Experimental group, *F* Females, *M* Males, *m* months, *PG* Placebo group, *Tx* treatment, *y* years

### Study characteristics

Gizani et al. [30] assessed the development of white spot lesions (WSL) on photographs [34], following the administration of lozenges containing *Lactobacillus reuteri* for a mean period of 17 months. The other three studies investigated gingival inflammation for a maximum period of 1 month, using gingivitis [33, 35] or bleeding [36] indices. The interventions assessed were lozenges with *Streptococcus salivarius* M18 only [29], lozenges containing *Streptococcus salivarius K12*, *Lactobacillus paracasei*, *Lactobacillus plantarum*, *Lactobacillus acidophilus* and *Lactobacillus reuteri* [31], as well as lozenges with *Lactobacillus reuteri* and a drink with *Lactobacillus casei* strain Shirota [32]. Three of the retrieved studies assessed adverse effects [29–31].

### Risk of bias within studies

Three studies were assessed to be of low risk of bias [29–31], while for Kohar et al. [32] most domains were considered to be of unclear risk (Table 3).

**Table 3:** Summary of the risk of bias assessment

Domain	Benic [2019] [29]	Gizani et al. [2016] [30]	Habib [2016] [31]	Kohar et al. [2015] [32]
**1**	Low	Low	Low	Unclear
**2**	Low	Unclear	Unclear	Unclear
**3**	Low	Low	Low	Unclear
**4**	Low	Low	Low	Unclear
**5**	Low	Low	Low	Low
**6**	Low	Low	Low	Low
**7**	Unclear	Unclear	Unclear	Unclear
**Summary**	**Low**	**Low**	**Low**	**Unclear**

Domains examined: 1: Random sequence generation 2: Allocation concealment, 3: Blinding of participants and personnel, 4: Blinding of outcome assessment, 5: Incomplete outcome data, 6: Selective outcome reporting, 7: Other potential threats to validity

### Results of individual studies

No statistically significant benefit was noted regarding the presence (*p* = 0.515) or the mean number of new WSL (*p* = 0.423) [30]. In addition, no statistically significant effect was demonstrated in terms of gingival inflammation (Benic et al. [29], *p* = 0.867; Habib [31], *p* = 0.797; Kohar et al. [32], *p* = 0.053). Finally, no adverse effects were noted in any of the included studies. Gizani et al. [30] reported that 8 participants could not tolerate the taste of the lozenges. Habib [31] reported one participant with gastrointestinal pain and diarrhoea, but it was later shown that this individual had received the placebo.

### Risk of bias across studies and additional analyses

Overall, the confidence in the obtained information was moderate (Table 4).

**Table 4:** Quality of available evidence

Quality assessment						No of patients		Effect	Quality
Studies	Risk of bias	Inconsistency	Indirectness	Imprecision	Other	Probiotic	Control		
**Enamel demineralization development**									
1	Not serious	Not serious	Not serious	Serious^1^	None	42	43	No difference	⨁⨁⨁◯ **Moderate**
**Gingival inflammation development**									
3	Not serious	Not serious	Not serious	Serious^a^	None	81	71	No difference	⨁⨁⨁◯ **Moderate**

^a^The number of patients analyzed was limited

## Discussion

### Summary of available evidence

Patients undergoing orthodontic treatment are linked with significant biofilm accumulation, thus exposing them to a greater risk of caries and gingivitis [37–39]. Nowadays, several reports have investigated probiotic effects in enhancing oral health in the general dental population, but, up to date, their effectiveness remains inconclusive [21, 22]. To the best of our knowledge, studies solely focusing on the clinical effects on orthodontic patients have not been previously summarized in an evidence-based manner.

Based on the data presented in this systematic review, there is a moderate level of evidence that administering probiotics to orthodontic patients does not have an effect on WSL development and gingival inflammation in the short-term, while no marked adverse effects were noted. Studies investigating the effect of probiotics on various microbiological parameters during orthodontic treatment have been conflicting [29–31, 40–43]. The lack of significant results presented in the current systematic review might be attributed to various causes, including the use of inappropriate and ineffective bacterial strains, ineffective concentrations of bacteria and administration protocols, ineffectiveness of the selected probiotic strain to colonize effectively the oral environment or strain inability to compete with the bacteria and biofilm accumulation present in the oral cavity [31].

Up to the present time, no consensus has been reached about which bacterial strain is most appropriate and effective. Some reports have found that the Lactobacillus species have shown positive effects in the treatment of periodontal diseases, including *Lactobacillus reuteri* strains [44–46], *Lactobacillus paracasei* [47], *Lactobacillus salivarius*, *Lactobacillus plantarum* and *Lactobacillus rhamnosus* [48–52]. *Lactobacillus brevis* has also been suggested to be potentially beneficial in view of its anti-inflammatory characteristics [53]. *Bifidobacterium* is another species that has been found to exert a positive impact on periodontal disease [54]. Based on our knowledge, the *Streptococcus salivarius K12* strain used by Habib [31], has not been previously assessed for its effect in treating gingivitis, but has been tested for changes in oral malodor parameters [55]. The *Streptococcus salivarius M18* strain has been mainly tested for anti-caries activity [56, 57].

In principle, multi-strain probiotics products could possess synergistic and symbiotic properties because of the interactions of each strain with the others. However, some very limited data suggests that probiotic strains may also exhibit inhibitory properties against each other. For instance, hydrogen peroxide and bacteriocin production may induce the desired effect when inhibiting endogenous strains such as *Streptococcus mutans*, while, simultaneously they might also disable other probiotic strains in the same formulation, thus reducing its effectiveness [58].

The concentrations required for producing the desired outcomes from oral probiotic formulations have not been widely investigated. It is critical to be certain about the exact dose required to initiate a dose-response reaction during the administration of probiotics. In the field of medicine, the industry standard for the counts of viable bacteria should range from 1 × 10^6^ to 1 × 10^9^ CFU [59]. However, when using oral probiotics, it is logical to assume that a lower dose or concentration would be required, since it does not have to pass through the gastrointestinal system. The vast majority of probiotic studies evaluating various oral health parameters have used concentrations of 1 × 10^8^ CFU. Moreover, it is important to remember that each individual strain has a different potential for oral colonization [31]. All doses should be selected according to the specific strain used.

In addition, the administration method and duration may also modulate the effect of a probiotic product. Various vehicles for oral probiotics have been employed, including gums, lozenges, tablets, drops and drinks [21, 22]. It has been suggested, for example, that the use of vehicles derived from milk that contain calcium, could potentially increase the anti-cariogenic effect [24]. Milk derived products produce also ammonia that helps increase pH and delay biofilm formation, by preventing bacterial adhesion on teeth [60].

Furthermore, effective probiotic activity, necessitates first adherence and subsequently colonization of oral surfaces [40]. These processes could be compromised in the case of a mature biofilm that is difficult to penetrate, or in the existence of an oral pH that is not compatible with bacterial viability [47]. Moreover, the capacity of a probiotic strain to colonize might vary between members of the same species, as it has been demonstrated for *Lactobacilli* [61–63]. Finally, there is the possibility that the administered strains are unable to compete with the quantity of the bacteria and plaque accumulation present in the oral cavity, as is possibly the case with orthodontic patients [47]. In such cases, higher concentrations of probiotics or administration for a longer duration may be required to demonstrate any potential for clinical improvements [31]. Supplementation with probiotics for periods 1 month or less as reported in the located studies may not be sufficient for the strain to colonize and establish a stable microbiome. Recently, alterations in bacterial composition was only detected after 6 weeks administration of an oral probiotic preparation [64].

Apart from factors associated with specific probiotic characteristics or the mode/duration of administration, patients’ compliance could also affect results. Although in the retrieved studies compliance was found to vary from good to excellent, these assessments were based on patient self-report [29–31]. Finally, the diet of participants during the interventions, the potential use of antibacterial or antiseptic products, changes in brushing/flossing technique and swallowing or chewing the lozenge rather than sucking it, thereby washing-out the probiotic from the mouth, could have affected the reported changes [31, 40].

### Strengths and limitations

For this review we adhered to well-established guidelines and focused on RCTs that provide the highest level of evidence in health care interventions. As far as we can know there has been no other review conducted on the possible effectiveness of probiotics on different clinical parameters in orthodontic patients. The search was extensive, comprehensive, without restrictions and every effort to reduce methodological bias was made.

The characteristics of the information located gave rise to some limitations as well. Due to insufficient information, it was not possible to conduct analyses for ‘small study effects’, publication bias or subgroup analyses. Moreover, the small number of individuals analysed could pose a threat to the precision of the results. Finally, the short duration of most interventions may confound the results and the use of specific strains, concentrations, dose regimens or modes of administration, might diminish the generalizability of the retrieved information.

### Recommendations for future research

The use of probiotics has been widely accepted by the general population by virtue of their natural source, as well as their beneficial effects on conditions pertaining to oral and overall health, like caries, periodontal tissues, nutrition status, immune response and respiratory infections [65–71]. However, further research is needed, in order to optimize probiotic use and quantify the extent of clinical benefit. In order to take full advantage of using oral probiotics, a more complete understanding regarding their mechanism of action in the area of adhesion and colonization and the capabilities of the different strains is required. Although nowadays, more and more research focus on the use of probiotics, the literature is still unable to reach a consensus on the optimum duration required or the ideal concentration or dose regimen and mode of administration for each probiotic strain. It is essential to understand the efficacy of each strain when used alone, as well as to evaluate the potential synergistic effects of combining probiotic strains into a single entity.

As orthodontic patients require continuous and rigorous oral hygiene control, caries prevention and maintenance of gingival health, more high-quality studies, involving different combinations of probiotic strains and of longer durations of intervention and follow-up are warranted. Moreover, instead of testing the use of probiotics to combat established gingivitis, research could be conducted on the possibility of preventing gingivitis using probiotics prior to the bonding of orthodontic brackets. Although much is known about probiotics in the gastrointestinal field, there is a great deal of knowledge to be learned pertaining to probiotics for oral health.

## Conclusions

Overall, probiotic administration does not seem to have an effect on the gingival inflammation and enamel decalcification development in patients under treatment with fixed orthodontic appliances. Further RCTs with particular focus on controlling the various possible sources of data, involving different combinations of probiotic strains and of longer duration of intervention and follow-up are required.

## Supplementary information

**Additional file 1: Table S1.** Eligibility criteria for the present systematic review. **Table S2.** Strategy for database search (up to September 1st, 2019). **Table S3.** Studies excluded during full-text eligibility assessment with reasons.

## Data Availability

All data and materials are available upon request.

## Abbreviations

PICOS: Participants, Interventions, Comparisons, Study design
PROSPERO: International prospective register of systematic reviews
RCT: Randomized controlled clinical trials
WSL: white spot lesions
