# Supplementary material for: Do probiotics promote oral health during orthodontic treatment with fixed appliances? A systematic review
Source: BMC Oral Health. 2020 Apr 25;20:126. doi: 10.1186/s12903-020-01109-3 (PMC7183645; doi:10.1186/s12903-020-01109-3)
Supplement: Supplementary file 1 — Additional file 1: Table S1. Eligibility criteria for the present systematic review. Table S2. Strategy for database search (up to September 1st, 2019). Table S3. Studies excluded during full-text eligibility assessment with reasons. [file 12903_2020_1109_MOESM1_ESM.docx]

**Do probiotics improve oral health during orthodontic treatment with fixed appliances? A systematic review [Supplementary material]**

**Supplementary Table 1.** Eligibility criteria for the present systematic review.

| **Domain** | **Inclusion criteria** | **Exclusion criteria** |
| --- | --- | --- |
| **Participants** | - Healthy individuals of any age undergoing orthodontic treatment with fixed appliances. | - Subjects with craniofacial anomalies or syndromes of the head and neck region. - Individuals with systematic diseases; individuals using antibiotics or antimicrobial agents |
| **Interventions** | - Probiotic treatments of any kind [with accurate description regarding the type of probiotic, regimen of administration, dosage, etc.]. |  |
| **Comparisons** | - Placebo intervention, no intervention or another probiotic intervention. |  |
| **Outcomes** | - Primarily, clinical measurements on gingival inflammation (e.g. bleeding on probing, gingival indices, etc.) and enamel demineralization development (e.g. white spot lesion indices, caries indices, etc.). - Secondarily, person reported outcomes (e.g. preferences, experiences, quality of life, satisfaction, etc.), as well as adverse effects and economic evaluation data. | - Plaque measurements were not considered, as reductions in plaque do not always directly reflect benefits in oral health, which is the primary goal of the intervention.^1^ - Non-clinical outcomes (e.g. oral microbial flora levels and composition). |
| **Study design** | - Randomized clinical trials. | - Animal studies. - Non-randomized studies, studies without control groups; non-comparative studies (case reports and case series). - Systematic reviews and meta-analyses. |

^1^American Dental Association. Acceptance program guidelines: adjunctive dental therapies for the reduction of plaque and gingivitis. Chicago, Illinois: American Dental Association, Council on Scientific Affairs, 2011.**Supplementary Table 2.** Strategy for database search (up to September 1st, 2019).

| **Database** | **Search strategy** | **Hits** |
| --- | --- | --- |
| **PubMed** | ((Probiot*) OR (Lactobacillus acidophilus) OR (ATCC 4356) OR (Bifidobacterium bifidum) OR (ATCC 29521) OR (Lactobacillus rhamnosus) OR SP1 OR (Streptococcus salivarius) OR (Lactobacillus plantarum) OR (Lactobacillus paracasei) OR (Lactobacillus reuteri) OR (Streptococcus uberis) OR (Streptococcus oralis) OR (Streptococcus rattus) OR (Bifidobacterium animalis)) AND ("fixed appliance" OR orthodon* OR "fixed orthodontic" OR bracket* OR multibracket) | **133** |
| **Cochrane Central Register of Controlled Trials** | ((Probiot*) OR (Lactobacillus acidophilus) OR (ATCC 4356) OR (Bifidobacterium bifidum) OR (ATCC 29521) OR (Lactobacillus rhamnosus) OR SP1 OR (Streptococcus salivarius) OR (Lactobacillus plantarum) OR (Lactobacillus paracasei) OR (Lactobacillus reuteri) OR (Streptococcus uberis) OR (Streptococcus oralis) OR (Streptococcus rattus) OR (Bifidobacterium animalis)) AND ("fixed appliance" OR orthodon* OR "fixed orthodontic" OR bracket* OR multibracket) in Title Abstract Keyword - (Word variations have been searched) | **2** |
| **Cochrane Database of Systematic Reviews** | ((Probiot*) OR (Lactobacillus acidophilus) OR (ATCC 4356) OR (Bifidobacterium bifidum) OR (ATCC 29521) OR (Lactobacillus rhamnosus) OR SP1 OR (Streptococcus salivarius) OR (Lactobacillus plantarum) OR (Lactobacillus paracasei) OR (Lactobacillus reuteri) OR (Streptococcus uberis) OR (Streptococcus oralis) OR (Streptococcus rattus) OR (Bifidobacterium animalis)) AND ("fixed appliance" OR orthodon* OR "fixed orthodontic" OR bracket* OR multibracket) in Title Abstract Keyword - (Word variations have been searched) | **0** |
| **Scopus** | TITLE-ABS((Probiot*) OR (Lactobacillus acidophilus) OR (ATCC 4356) OR (Bifidobacterium bifidum) OR (ATCC 29521) OR (Lactobacillus rhamnosus) OR SP1 OR (Streptococcus salivarius) OR (Lactobacillus plantarum) OR (Lactobacillus paracasei) OR (Lactobacillus reuteri) OR (Streptococcus uberis) OR (Streptococcus oralis) OR (Streptococcus rattus) OR (Bifidobacterium animalis)) AND ("fixed appliance" OR orthodon* OR "fixed orthodontic" OR bracket* OR multibracket) AND (LIMIT-TO (SUBJAREA,"DENT")) | **173** |
| **Web of Science™ Core Collection** | TOPIC: (((Probiot*) OR (Lactobacillus acidophilus) OR (ATCC 4356) OR (Bifidobacterium bifidum) OR (ATCC 29521) OR (Lactobacillus rhamnosus) OR SP1 OR (Streptococcus salivarius) OR (Lactobacillus plantarum) OR (Lactobacillus paracasei) OR (Lactobacillus reuteri) OR (Streptococcus uberis) OR (Streptococcus oralis) OR (Streptococcus rattus) OR (Bifidobacterium animalis)) AND ("fixed appliance" OR orthodon* OR "fixed orthodontic" OR bracket* OR multibracket)) Timespan: All years. Databases: WOS, KJD, RSCI, SCIELO, ZOOREC. Search language=Auto | **70** |
| **Arab World Research Source** | TI (probiotic* AND orthodontic*) OR AB (probiotic* AND orthodontic*) | **0** |
| **ClinicalTrials.gov** | Orthodontic \| probiotics | **3** |
| **ProQuest Dissertations and Theses Global** | ti(((Probiot*) OR (Lactobacillus acidophilus) OR (ATCC 4356) OR (Bifidobacterium bifidum) OR (ATCC 29521) OR (Lactobacillus rhamnosus) OR SP1 OR (Streptococcus salivarius) OR (Lactobacillus plantarum) OR (Lactobacillus paracasei) OR (Lactobacillus reuteri) OR (Streptococcus uberis) OR (Streptococcus oralis) OR (Streptococcus rattus) OR (Bifidobacterium animalis)) AND ("fixed appliance" OR orthodon* OR "fixed orthodontic" OR bracket* OR multibracket)) OR ab(((Probiot*) OR (Lactobacillus acidophilus) OR (ATCC 4356) OR (Bifidobacterium bifidum) OR (ATCC 29521) OR (Lactobacillus rhamnosus) OR SP1 OR (Streptococcus salivarius) OR (Lactobacillus plantarum) OR (Lactobacillus paracasei) OR (Lactobacillus reuteri) OR (Streptococcus uberis) OR (Streptococcus oralis) OR (Streptococcus rattus) OR (Bifidobacterium animalis)) AND ("fixed appliance" OR orthodon* OR "fixed orthodontic" OR bracket* OR multibracket)) in Full Text | **20** |

**Supplementary Table 3.** Studies excluded during full-text eligibility assessment with reasons.

| **Study** | **Reason for exclusion** |
| --- | --- |
| Alp S, Baka ZM. Effects of probiotics on salivary Streptecoccus mutans and Lactobacillus levels in orthodontic patients. Am J Orthod Dentofacial Orthop 2018;154:517-523. | Non-clinical measurements. |
| Benic GZ. Biofilm management with oral probiotics in patients with fixed orthodontic appliances. Thesis. Doctor of Clinical Dentistry (Orthodontics). University of Otago, 2016 | Thesis on which a later published paper was based. |
| ClinicalTrials.gov Identifier: NCT01657539: Effect of Probiotics Containing Yogurts on the Composition of Biofilms in Patients Under Orthodontic Treatment. | Non-clinical measurements. |
| ClinicalTrials.gov Identifier: NCT02357771: Probiotic Lozenge Reduce Streptococcus Mutans in Plaque in Orthodontic Bracket Patients. | Non-clinical measurements. |
| ClinicalTrials.gov Identifier: NCT03004196: Comparison of Efficacy Of Probiotic Toothpaste and Chlorhexidine Mouthwash To Reduce S.Mutans. | Non-clinical measurements. |
| Goyal N, Shamanna PU, Varughese ST, Abraham R, Antony B, Emmatty R, Paul P. Effects of amine fluoride and probiotic mouthwash on levels of Porphyromonas gingivalis in orthodontic patients: A randomized controlled trial. J Indian Soc Periodontol 2019;23:339-344. | Non-clinical measurements. |
| JIvraj, FE. Effectiveness of the Lorodent Probiotic Lozenge in Reducing Plaque and S. Mutans Levels in Orthodontic Patients: A Randomized, Double-Blind, Placebo- Controlled Trial. MSc Thesis. University of Toronto, 2015a | Plaque measurements; non-clinical measurements. |
| Jose JE, Padmanabhan S, Chitharanjan AB. Systemic consumption of probiotic curd and use of probiotic toothpaste to reduce Streptococcus mutans in plaque around orthodontic brackets. Am J Orthod Dentofacial Orthop 2013;144:67-72. | Non-clinical measurements. |
| Megha S, Shalini G, Varsha SA, Abhishek D, Neetu J. Effect of Short-Term Placebo-Controlled Consumption of Probiotic Yoghurt and Indian Curd on the Streptococcus mutans Level in Children Undergoing Fixed Interceptive Orthodontic Therapy. Turk J Orthod 2019;32:16-21. | Non-clinical measurements. |
| Pinto GS, Cenci MS, Azevedo MS, Epifanio M, Jones MH. Effect of yogurt containing Bifidobacterium animalis subsp. lactis DN-173010 probiotic on dental plaque and saliva in orthodontic patients. Caries Res 2014;48:63-8. | Non-clinical measurements. |
| Shah SS, Nambiar S, Kamath D, Suman E, Unnikrishnan B, Desai A, Mahajan S, Dhawan KK. Comparative evaluation of plaque inhibitory and antimicrobial efficacy of probiotic and chlorhexidine oral rinses in orthodontic patients: a randomized clinical trial. Int J Dent 2019;2019:1964158. | Intervention not described accurately [regarding the type of probiotic, regimen of administration, dosage, etc.]. Authors contacted but no response received. |
